# A 10-year review of isoniazid-resistant TB management in Uzbekistan 2009-2020

**DOI:** 10.5588/ijtldopen.23.0533

**Published:** 2024-07-01

**Authors:** M.L. Rekart, P. Thit, M. Oluya, S. Moe, T. Hasan, N. Parpieva, K. Safaev, A. Khristusev, T. Zinaida, J. Singh, S. Allamuratova, I. Azamat, C.G. Restrepo, N. Sitali, J. Achar, J.L. Alvaraez, A. Sinha

**Affiliations:** ^1^ Médecins Sans Frontières (MSF), Amsterdam, The Netherlands;; ^2^ MSF, Tashkent, Uzbekistan;; ^3^ Republican Specialized Scientific and Practical Medical Center of Tuberculosis and Pulmonology, Tashkent, Uzbekistan;; ^4^ Republican Center of Tuberculosis and Pulmonology, Nukus, Uzbekistan;; ^5^ MSF, Berlin, Germany;; ^6^ Department of Global Public Health, Karolinska Institutet, Stockholm, Sweden;; ^7^ MSF, London, UK

**Keywords:** INH resistance, Central Asia, 6REZLfx, drug-resistant TB, DR-TB

## Abstract

**BACKGROUND:**

Isoniazid (INH, H) resistance is the most common drug-resistant TB pattern, with treatment success rates lower than those in drug-susceptible TB. The WHO recommends a 6-month regimen of rifampicin (RIF, R), ethambutol (EMB, E), pyrazinamide (PZA, Z), and levofloxacin (Lfx) (6REZLfx) for INH-resistant, RIF-susceptible TB (H^R^R^S^-TB). Uzbekistan has a high burden of TB (62/100,000 population) and multidrug-resistant TB (12/100,000 population).

**METHODS:**

We conducted a retrospective, descriptive study of microbiologically confirmed H^R^R^S^-TB using routinely collected programmatic data from 2009 to 2020.

**RESULTS:**

We included 854 H^R^R^S^-TB cases. Treatment success was 80.2% overall. For REZLfx, the treatment success rate was 92.0% over a short treatment duration, with no amplifications to RIF or second-line anti-TB drug resistance. We documented 46 regimens with REZLfx plus linezolid (success 87.0%) and 539 regimens using kanamycin or capreomycin (success 76.6%). We identified 37 treatment failures (4.3%), 30 deaths (3.5%), 25 resistance amplifications (2.9%), including eight to RIF (0.9%), and 99 lost to follow-up (LTFU) cases (11.6%). Unsuccessful outcomes were more common with older age, diabetes, chest X-ray cavities, smear positivity, smear-positive persistence, and male sex. LTFU was more common with injection-containing regimens.

**CONCLUSIONS:**

REZLfx is a safe and effective first-line treatment for INH-resistant, RIF-susceptible TB. Treatment success was lower and LTFU was higher for injection-containing regimens.

## RÉSUMÉ

**CONTEXTE:**

La résistance à l'isoniazide (INH, H) est la forme de TB pharmacorésistante la plus courante, avec des taux de réussite thérapeutique inférieurs à ceux de la TB pharmacosensible. L'OMS recommande un traitement de six mois à base de rifampicine (RIF, R), d'éthambutol (EMB, E), de pyrazinamide (PZA, Z) et de lévofloxacine (LFx) (6REZLfx) pour la TB résistante à l'INH et sensible au RIF (H^R^R^S^-TB). En Ouzbékistan, la prévalence de la TB est élevée, avec un taux de 62 cas pour 100 000 habitants, ainsi que de la TB multirésistante, avec un taux de 12 cas pour 100 000 habitants.

**MÉTHODES:**

Une étude rétrospective et descriptive de la H^R^R^S^-TB confirmée microbiologiquement a été réalisée en utilisant des données programmatiques collectées de manière routinière de 2009 à 2020.

**RÉSULTATS:**

Nous avons inclus 854 cas de H^R^R^S^-TB. Le taux de réussite du traitement global était de 80,2%. Pour le traitement avec REZLfx, le taux de réussite était de 92,0% sur une courte durée, sans résistance au RIF ni aux médicaments antituberculeux de deuxième ligne. Nous avons observé 46 schémas thérapeutiques associant REZLfx et linézolide avec un taux de réussite de 87,0%, ainsi que 539 schémas thérapeutiques utilisant la kanamycine ou la capréomycine avec un taux de réussite de 76,6 %. Nous avons enregistré 37 échecs thérapeutiques (4,3%), 30 décès (3,5%), 25 cas de résistance amplifiée (2,9%), dont huit au RIF (0,9%), et 99 cas de perte de suivi (LTFU, pour l’anglais « *loss to follow-up* ») (11,6%). Les échecs étaient plus fréquents chez les patients âgés, diabétiques, présentant des cavités à la radiographie thoracique, un frottis positif persistant et de sexe masculin. La prolongation de la durée d'utilisation était plus fréquente avec les schémas contenant des injections.

**CONCLUSIONS:**

REZLfx est un traitement de première intention sûr et efficace contre la TB résistante à l'INH et sensible aux RIF. Le succès du traitement était plus faible et le nombre de LTFU était plus élevé pour les schémas contenant des injections.

Isoniazid (INH, H) resistance in *Mycobacterium tuberculosis* (MTB) is likely a natural phenomenon due to frequent spontaneous mutations, especially at codon 315 of the *kat*G gene resulting in moderate- to high-level resistance and the c-15t mutation in the *inh*A promoter region resulting in low-level resistance.^1,2^ INH resistance is the most common TB resistance pattern, accounting for over 1 million new cases annually.^3,4^ Furthermore, INH monoresistance is the most common TB monoresistance pattern.^5,6^

INH-resistant, rifampicin (RIF, R) susceptible TB (H^R^R^S^-TB) includes strains susceptible and resistant to other first-line drugs, i.e., INH-monoresistant and polydrug-resistant TB (PDR-TB), respectively. However, first-line susceptibility results are often incomplete in H^R^R^S^-TB cases, except for INH and RIF. The global average annual incidence of H^R^R^S^-TB among all TB cases from 2002 to 2016 was 8.5% (95% confidence interval [CI] 7.4–9.7) overall, 7.3% (95% CI 6.1–8.6) for new cases, and 14.0% (95% CI 12–17) for previously treated cases.^7^ H^R^R^S^-TB incidence is increasing among persons incarcerated or homeless.^5,8–10^

The global prioritised use of Xpert^®^ MTB/Rif assays (Cepheid, Sunnyvale, CA, USA) means that RIF-susceptible TB cases are usually treated for drug-susceptible TB (DS-TB) without INH resistance testing, resulting in RIF monotherapy during the continuation phase for INH resistance cases.^3,11^

Treatment success with INH resistance is often poor compared with DS-TB, with success rates of 61.0–85.0%^3,4,6^ and more frequent adverse events.^4^ Failure rates range from 0.0–45.0% and mortality from 1.9% to 14.2%.^3,4,6^ Poor outcomes are associated with HIV co-infection,^3,6^ longer treatment duration,^12^ streptomycin use,^12^ age,^6^ male sex,^3,6^ previous TB treatment,^12^ cavitary disease,^12^ smear positivity,^4^ and smear-positive persistence.^12^

Historically, H^R^R^S^-TB treatment regimens were not standardised,^3,11^ and access to drug susceptibility testing (DST) was limited, often focusing on RIF-resistant TB (RR-TB).^3,13^ The choice of regimen commonly depended on the previous treatment history and provider preference, often including a fluoroquinolone (FQ) and/or an aminoglycoside for 9–12 months.^11,12^

In 2018, the WHO issued new treatment recommendations for H^R^R^S^-TB, replacing 9 months of RIF, ethambutol (EMB, E), and pyrazinamide (PZA, Z) with 6 months of REZ plus levofloxacin (Lfx).^11,12^ The WHO supports the addition of high-dose INH for strains with *inh*A but not *kat*G mutations.^12^

Globally, 64.3–78.6% of observed phenotypic INH resistance is associated with *kat*G315 mutations alone, 6.8–19.2% with *inh*A-15 mutations alone, and 14.6% with both.^14,15^ The WHO European region, including Uzbekistan, has high rates of H^R^R^S^-TB, INH resistance, and *kat*G315 mutations.^14,15^ One study identified *kat*G315 mutations in 94% of INH-resistant isolates in six ex-Soviet states, including Kazakhstan, which borders Karakalpakstan.^14^

H^R^R^S^-TB treatment can result in acquired resistance to RIF and second-line TB drugs. Acquired RIF resistance occurred in 44/1,160 (3.8%) H^R^R^S^-TB patients not treated with FQs and 1/221 (0.5%) treated with FQs in one review.^12^

Because of this changing landscape, reviews of H^R^R^S^-TB treatment outcomes within long-running, comprehensive programmes are important in overall TB management.

## Objectives

Our primary objective was to describe H^R^R^S^-TB treatment outcomes. Our secondary objectives were to 1) evaluate risk factors for unfavourable outcomes, 2) delineate treatment outcomes vis-a-vis treatment duration, 3) determine culture conversion rates, and 4) document frequencies of INH mutations.

## METHODS

### Study design

This is a retrospective, observational study of microbiologically confirmed H^R^R^S^-TB cases in Karakalpakstan, Uzbekistan, using routinely collected programmatic data.

### Setting

Karakalpakstan is an autonomous republic in north-western Uzbekistan (land area 166,600 km^2^, population 1.88 million) with 16 rayons (districts) and one city, Nukus, the capital, where Médecins Sans Frontières (MSF) supports a centralised laboratory allowing for standardised, high-quality data. MSF has worked with the Ministry of Health (MOH) to strengthen TB diagnosis and treatment since 1998, using locally developed guidelines based on WHO recommendations. In 2009, MSF started supporting MOH drug-resistant TB (DR-TB) management in four rayons, expanding to all rayons and including drug-susceptible TB (DS-TB) by 2020. Approximately three-quarters of DR-TB and half of DS-TB cases were enrolled in an MSF-supported programme by 2020.

Karakalpakstan TB guidelines include management and follow-up by a multidisciplinary team (doctors, nurses, and social and mental health workers), social support, ancillary drugs for side effects, self-administered therapy (SAT) for stable patients on injection-free, first-line regimens, and directly observed treatment (DOT) for complicated patients and those on injection-containing and/or second-line regimens. Monthly bacteriologic monitoring of positive TB cultures and a 2-year follow-up for relapse are recommended.

Treatment regimens and regimen changes are decided by a medical advisory committee of local TB experts, the TB Consilium. They review each case and prescribe treatment based on national TB treatment guidelines. Although clinical judgement is involved, they operate within the national guidelines, and all patients are treated within the national programme. Therefore, most H^R^R^S^-TB patients are started on similar treatment. From 2009 to 2018, the recommended treatment for H^R^R^S^-TB was REZ for 9 months. The recommendation was changed to REZLfx for 6 months in 2019.

MSF support focuses on health education, adherence counselling, and help with patients who have treatment challenges via phone calls, home visits, and counselling during clinic visits. All patients diagnosed with TB and initiated on treatment in an MSF-supported rayon are eligible for MSF support.

### Variables

We collected data on demographics, HIV, diabetes, TB contact, previous TB treatment, previous INH exposure, smear, culture, DST, GenoType™ MTBDR*plus* (Hain Lifescience, Nehren, Germany), chest X-ray (CXR), date of treatment commencement and duration, regimen, regimen change, outcome, and drug side effects.

### Participants

All patients testing positive for TB at the MSF-supported Republican Center of Tuberculosis and Pulmonology Hospital Laboratory (Nukus, Karakalpakstan, Uzbekistan) from March 2009 through December 2020 and enrolled in an MSF-supported programme were eligible. We have no detailed data on non-enrolled patients. We defined H^R^R^S^-TB as TB cases with bacteriologic results confirming phenotypic or genotypic INH resistance and RIF susceptibility without known resistance to second-line TB drugs. We excluded unenrolled patients and/or those with a positive Xpert MTB/Rif assay only.

### Definitions

We used WHO definitions for H^R^R^S^-TB, previous treatment, smear-positive persistence, culture conversion/reversion, transferred-out, and treatment outcomes.^16^ We defined amplified resistance as H^R^R^S^-TB cases that failed treatment and were found to be resistant to RIF and/or second-line anti-TB drugs.

### Data sources and measurements

Epidemiological TB data in Karakalpakstan were collected by the MOH and MSF and transferred to Epi Info (Centers for Disease Control and Prevention, Atlanta, USA). Only study-relevant data were recorded, and access was restricted to the investigators.

The Figure shows the testing algorithm used in the MSF-supported Karakalpakstan Mycolab BSL3 Laboratory for INH resistance. The use of the pDST BD BACTEC™ MGIT™ 960 SIRE kit with the BACTEC MGIT 960 (BD, Franklin Lakes, NJ, USA) at an INH drug critical concentration of 1.0 mg/L started in 2005. INH resistance was also identified using the MTBDR*plus* v2.0 line-probe assay to detect mutations in the *kat*G and *inh*A genes beginning in 2010. Annual external quality assessment is provided by the WHO Supranational Reference Laboratory in Gauting, Germany. Batch testing for the SIRE drug kit and the MTBDR*plus* kit used the sensitive control strain H37Rv and a known resistant strain.

**Figure.**
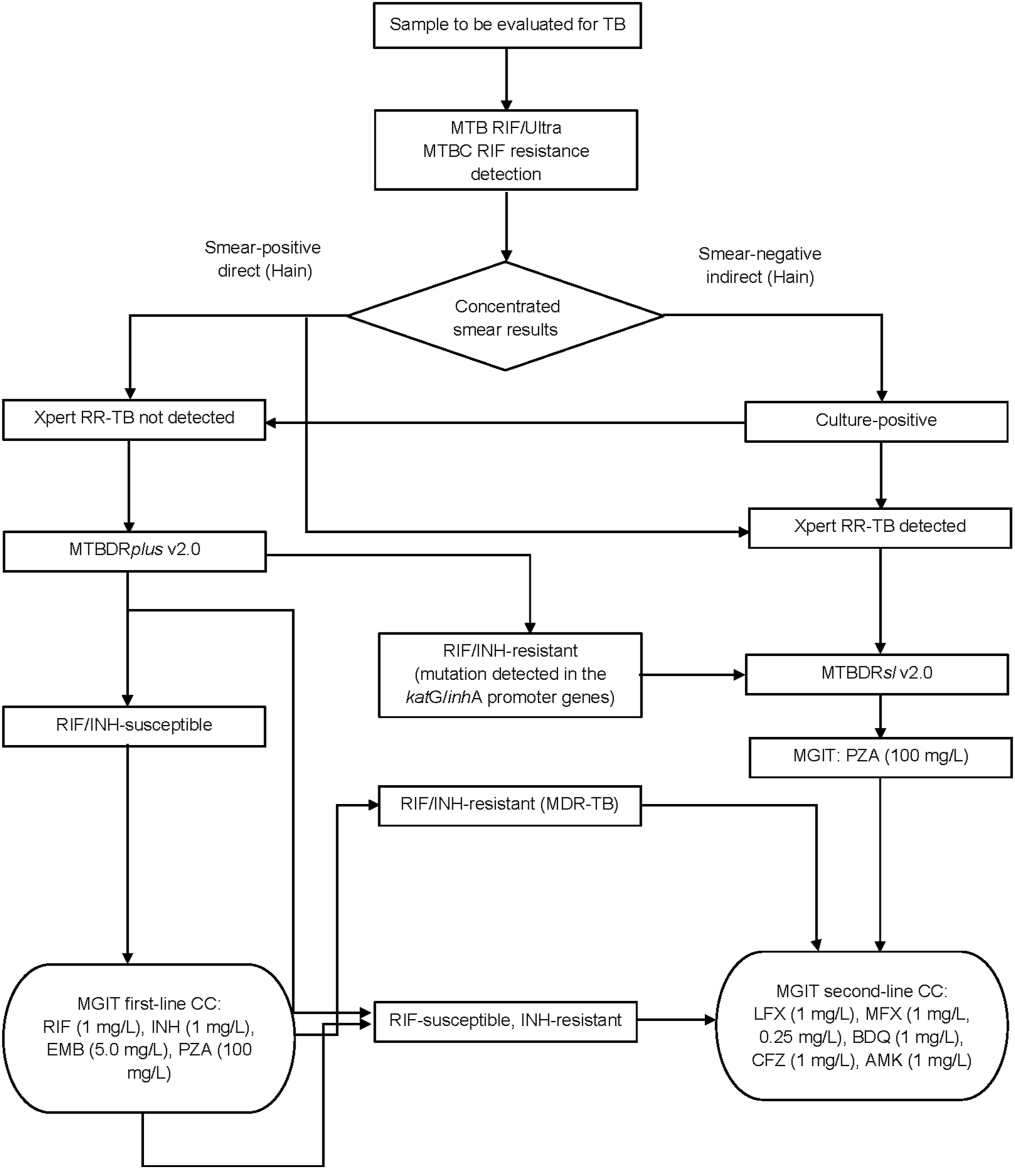
Testing algorithm for the identification of INH resistance. *RIF CC changed in 2021 to 0.5 mg/L. MTB = *Mycobacterium tuberculosis*, RIF = rifampicin; MTBC = MTB complex; RR-TB = rifampicin-resistant TB; INH = isoniazid; MDR-TB = multidrug-resistant TB; MGIT = Mycobacteria Growth Indicator Tube; CC = critical concentration; EMB = ethambutol; PZA = pyrazinamide; LFX = levofloxacin; MFX = moxifloxacin; BDQ = bedaquiline; CFZ = clofazimine; AMK = amikacin.

Because of algorithm changes and inconclusive results, not all 2009–2020 H^R^R^S^-TB cases had susceptibility results for all first-line drugs other than INH and RIF. Routine *kat*G and *inh*A testing began in 2017, and second-line testing for resistance was initiated in 2019.

### Quantitative analysis and statistical methods

We report descriptive analyses of baseline characteristics, interim responses, and end-of-treatment outcomes. For treatment outcome rates, the entire cohort was used as the denominator. χ^2^ statistics with Yates correction were used with one degree of freedom and a 0.05 significance level.

### Ethical considerations

This study fulfilled the exemption criteria set by the MSF Ethics Review Board for a posteriori analyses of routinely collected clinical data and did not require MSF ERB review. This study was conducted with permission from the MSF Operational Centre Amsterdam, The Netherlands.

## RESULTS

Of the 11,760 potentially eligible participants, we included 854 enrolled H^R^R^S^-TB cases. Table 1 shows their baseline characteristics. Most were male (55.7%), smear-positive (62.9%), with a history of TB treatment (62.3%), and with a history of INH exposure (51.3%).

**Table 1.** Baseline characteristics of 854 enrolled (laboratory-confirmed) H^R^R^S^-TB cases, 2009–2020.

	Yes	No	Yes %
History of diabetes	84	739	10.2
Positive HIV test	3	706	0.4
History of TB contact	177	677	20.7
Abnormal CXR	854	0	100.0
CXR cavities	392	450	46.6
Positive TB smear	521	307	62.9
Previous TB treatment ≥1 month	532	322	62.3
Previous INH exposure ≥1 month	438	416	51.3
Positive TB culture	821	27	96.1
Male	475	379	55.7
Age, years			
<5	0	854	0.0
5–14	7	847	0.8
15–24	113	741	13.2
25–64	623	231	73.0
≥65	111	743	13.0

H^R^R^S^-TB = INH-resistant, rifampicin-susceptible TB; INH = isoniazid, CXR = chest X-ray.

### Primary objective: Treatment outcomes

H^R^R^S^-TB treatment success overall was 80.2% (685/854) (Table 2). The highest success rate was 92.0% (23/25) for REZLfx alone; however, this was a small, select group. Only one patient was ≥65 years old, and 23 were enrolled in 2019-2020. The success rate for REZ alone was 87.1% (202/232). REZLfx + linezolid (Lzd) alone was successful in 87.0% (40/46); however, this was a select cohort with only three patients ≥65 years (6.5%) and all enrolled in 2019-2020. Injection-free regimen success was 85.5% (265/310) vs. 76.8% (418/544) for injection-containing regimens, including 76.6% (413/539) with kanamycin (KM) or capreomycin (CPM). We found no statistically significant difference in successful outcomes between the regimens. We did not identify any relapses.

**Table 2.** Isoniazid-resistant, rifampicin-susceptible TB treatment outcomes by regimen.

Regimen	Patients (*n*)	Successful outcomes^16^ (*n*)	Success rate (%)	Treatment duration for successful outcomes, months Median [IQR]
All regimens	854	685	80.2	10.1 [9.4–11.7]
REZLfxCpm/Km	415	328	79.0	10.4 [9.7–12.0]
REZ alone	232	202	87.1	9.2 [9.0–10.1]
REZLfxCpm/Km-Pth	63	48	76.2	11.8 [10.5–12.8]
REZLfxLzd alone	46	40	87.0	9.4 [9.0–10.3]
REZLfx alone	25	23	92.0	6.6 [6.0–7.4]
Regimen with REZ	783	640	81.7	10.1 [9.2–11.5]
Regimens with REZLfx	552	444	80.4	10.4 [9.9–11.9]
Regimens with Km or Cpm	539	413	76.6	10.6 [9.9–12.2]
Injection-containing regimens*	544	418	76.8	10.6 [9.9–12.2]
Injection-free regimens	310	265	85.5	9.2 [8.9–10.1]

* Includes four regimens with streptomycin and 1 regimen with amikacin.

IQR = interquartile range; R = rifampicin; E = ethambutol; Z = pyrazinamide; Cpm = capreomycin; Lfx = levofloxacin; Km = kanamycin; Pth = prothionamide; Lzd = linezolid.

### Secondary Objective 1: Risk factors for unsuccessful outcomes

One hundred and sixty-nine patients (169/854, 19.8%) did not achieve a successful outcome, including 37 failures (4.3%, 37/854), 30 deaths (3.5%, 30/854), and 99 LTFU cases (99/854, 11.6%) (Table 3). Three patients were transferred out before the treatment outcome. Compared with all H^R^R^S^-TB patients, failure was more common with age ≥65 years (16.2% vs 13.0%), diabetes (24.3% vs 10.2%), smear positivity (83.8% vs 62.9%), and smear-positive persistence (55.6% vs 7.2%). Failure was less common with REZLfx alone (0.0% vs 2.9%), age 15-24 years (8.3% vs 13.2%), and initial culture positivity (40.5% vs 96.1%).

**Table 3.** H^R^R^S^-TB: distribution and risk factors for unfavourable treatment outcomes.*

	All treatment failure *n* (%)	Treatment failure plus amplification *n* (%)	Death *n* (%)	Lost to follow-up *n* (%)	All H^R^R^S^-TB *n* (%)
Total (rate)	37 (4.3)	25 (2.9)	30 (3.5)	99 (11.6)	854 (100.0)
Male	21 (56.7)	14 (56.0)	23 (76.7)	64 (64.6)	475 (55.7)
Age, years					
<5	0 (0.0)	0 (0.0)	0 (0.0)	0 (0.0)	0 (0.0)
5–14	1 (2.7)	1 (4.0)	0 (0.0)	1 (1.0)	7 (0.8)
15–24	3 (8.3)	1 (4.0)	0 (0.0)	7 (7.0)	113 (13.2)
25–64	27 (75.0)	19 (76.0)	22 (73.3)	77 (77.8)	623 (73.0)
≥65	6 (16.2)	4 (16.0)	8 (26.7)	14 (14.0)	111 (13.0)
Diabetes	9 (24.3)	6 (24.0)	3 (10.0)	14 (14.1)	84 (10.2)
HIV-positive	0 (0.0)	0 (0.0)	1 (3.3)	0 (0.0)	3 (0.4)
CXR cavities	20 (54.1)	12 (48.0)	16 (53.3)	55 (55.6)	392 (46.6)
Previous TB treatment ≥1 month	27 (73.0)	17 (68.0)	18 (60.0)	67 (67.7)	532 (62.3)
Previous INH exposure ≥1 month	17 (45.9)	14 (56.0)	15 (50.0)	56 (56.6)	438 (51.3)
Any REZ	32 (86.5)	25 (100)	21 (70.0)	85 (85.6)	795 (93.1)
Any aminoglycoside	22 (59.5)	11 (44.0)	21 (70.0)	79 (79.8)	543 (63.6)
Any Lfx	22 (59.5)	11 (44.0)	23 (76.7)	86 (86.9)	609 (71.3)
REZ-Cpm/Km-Lfx	14 (37.8)	7 (28.0)	14 (46.7)	60 (60.6)	412 (48.2)
REZ-Lfx alone	0 (0.0)	0 (0.0)	1 (3.3)	1 (1.0)	25 (2.9)
REZ-Lfx-Lzd	1 (2.8)	0 (0.0)	1 (3.4)	4 (4.0)	46 (5.4)
Injection-free regimens	15 (40.5)	14 (56.0)	9 (30.0)	20 (20.2)	310 (36.3)
Injection-containing regimens	22 (59.5)	11 (44.0)	21 (70.0)	79 (79.8)	544 (63.7)
Initial smear positivity	31 (83.8)	20 (80.0)	13 (43.3)	61 (61.6)	521 (62.9)
Smear-positive persistence	20 (54.1)	13 (52.0)	11 (36.7)	18 (18.2)	61 (7.2)
Initial positive culture	15 (40.5)	8 (32.0)	12 (40.0)	97 (98.0)	821 (96.1)

* Not including the three patients who were transferred out before a treatment outcome.

H^R^R^S^-TB = isoniazid-resistant, rifampicin-sensitive TB; INH = isoniazid; CXR = chest X-ray; R = rifampicin; E = ethambutol; Z = pyrazinamide; Cpm = capreomycin; Km = kanamycin; Lfx = levofloxacin; LZD = linezolid.

Amplified resistance occurred 25 times (2.9%, 25/854) among the failures. Twenty-four showed amplified resistance to KM; 12 were also CPM-resistant, including one resistant to moxifloxacin. Eight KM-resistant isolates acquired resistance to ≥1 first-line drugs, including RIF (8), EMB (4), and PZA (6). Acquired RR-TB cases were reclassified as MDR-TB (8/854, 0.9%). There were no amplifications on REZLfx alone or REZLfxLzd alone, although the numbers were small.

A higher proportion of deaths were male (76.7% vs. 55.7%), ≥65 years old (26.7% vs. 13.0%), and persistent smear-positive (36.7% vs. 7.2%). Fewer were 15-24 years old (0.0% vs. 13.2%) and initially culture-positive (40.0% vs. 96.1%). We found no association with the treatment regimen.

Compared with all H^R^R^S^-TB patients, a higher proportion of the 99 LTFU patients took an injectable agent (79.8% vs. 63.7%) and were persistently smear-positive (18.2% vs. 7.2%). Injection-free regimens were protective against loss to follow-up (20.0% injection-free LTFU vs. 79.8% injection-containing LTFU). We found no relationship to the year of diagnosis or to rural vs. city residence. We have no information on measures for patient support of individual LTFU cases.

### Secondary objective 2: Treatment outcomes vis-à-vis treatment duration

We have treatment durations on 98.7% of patients (843/854) (Table 2). The median duration for cured and completed patients was 10.1 months. The durations were shorter for injection-free regimens, especially REZLfx (6.6 months). The median duration to failure was 6.1 months, failure with amplification 5.1 months, death 4.2 months, and LTFU 4.4 months.

### Secondary objective 3: Culture conversion

We had 821 positive cultures from 854 patients (96.1%). Overall, 618 cultures converted (75.3%, 618/821): 616 (75.0%, 616/821) within 6 months and 476 (56.9%, 476/821) within 2 months. The median time to culture conversion (MTCC) was 33 days (interquartile range [IQR] 23–60). There were no culture reversions.

### Secondary objective 4: INH mutations

We found *kat*G and/or *inh*A mutations in 64.6% (175/271) of samples with both results. *kat*G was found in 85.7% overall (150/175) and alone in 80.6% (141/175). *inh*A was found in 19.4% overall (34/175) and alone in 14.3% (25/175). Both were found in 5.1% (9/175).

### Drug side effects

Drug side effects were uncommon, mild, and frequently associated with PZA (37/89 episodes, 41.6%). Twelve patients on EMB (recommended dosage 15–20 mg/kg/day) experienced ophthalmologic side effects, six on PZA developed hepatitis, and two on RIF or CPM had decreased creatinine clearance.

## DISCUSSION

We found a high treatment success rate of 92.0% (23/25) with one LTFU, one death, and no amplified resistance in a small, select group treated with ≥6REZLfx alone for a median duration of 6.6 months. Comparisons to the literature are complicated by inconsistencies in the inclusion/exclusion of patients who were LTFU, defaulted, transferred out, and died from other causes. However, a comparison based on ‘assessable’ regimen success, including cure, completion, failure, and death during treatment, is feasible. Our assessable treatment success rate of 95.8% (23/24, not including 1 LTFU) for ≥6REZLfx is comparable to the following assessable H^R^R^S^-TB treatment success rates: 97.6% (245/251) for ≥6(H)REZ-quinolone with or without INH (Fregonese et al.);^17^ 94.2% (65/69) utilising ≥6 months of RIF, FQ and various combinations of H, E, Z and Lzd (1 case) (Edwards et al.);^18^ 90.0% (18/20) for ≥6REZLfx (Wilson et al.);^19^ and 97.3% (73/75) for REZ-quinolone with a mean treatment duration of 9.1 months (Lee et al.^20^). Finally, Bang et al.^21^ reported H^R^R^S^-TB treatment success of 90.0% (36/40) for similar regimens to Edwards et al.^18^ with a mean duration of 7.6 months, including defaulted and transferred-out patients. Garcia et al.^3^ reported an H^R^R^S^-TB treatment success rate of 79.1% (626/791) for 9REZLfx, including a high LTFU rate (19.6%). These results are comparable to our overall success rate of 92.0% (23/25) for the full ≥6REZLfx cohort.

Our overall treatment success rate was comparable to others (80.2% vs. 77.2–83.0%).^3,19,21,22^ Many studies have shown lower H^R^R^S^-TB treatment success rates than DS-TB.^5,6,11,17,23–27^ In Karakalpakstan, DS-TB treatment success from 2017 to 2020 was 80.5% (5,582/6,931) (personal communication, K. J. Kudaybergenov, Director, MOH of the Republic of Karakalpakstan). H^R^R^S^-TB treatment success was not worse than local DS-TB cases (80.2% vs. 80.5%), potentially due to MSF support for H^R^R^S^-TB patients.

We documented 539 H^R^R^S^-TB patients treated with KM or CPM. The WHO guidelines state that there are no data on these injectable agents in H^R^R^S^-TB treatment.^12^ We found a lower treatment success rate with injection-containing versus injection-free regimens (76.8% vs. 85.5%) and a higher frequency of LTFUs (79.8% vs. 20.0%). Our results support recommendations to phase out injection-containing regimens.^3,17,28^

We documented a small, select cohort of 46 patients treated with REZLfxLzd alone. Only one case was found in the literature.^18^ The treatment success (87.0%) and median treatment duration (9.4 months) were similar to those of REZ alone.

### Unsuccessful outcomes

For unsuccessful outcomes, our findings were similar to those of other studies: mortality 3.5% vs. 1.9–4.8%,^3,17,18,21^ treatment failure 4.3% vs. 1.2–5.8%,^3,17,18,21^ LTFU 11.6% vs. 3.8%–19.6%,^3,17,18,21^ and acquired RR-TB 0.9% vs. 1.0% and 3.9%.^12,17,18^

### Treatment success factors

Our findings confirm previous research that treatment success was lower with age ≥65 years,^5,6,18^ diabetes,^5,10^ previous TB treatment,^5,11,23,29^ initial smear positivity,^6^ smear-positive persistence,^10,11^ and CXR cavities.^11^

### Side effects

We confirmed that PZA is the drug most often implicated in side effects.^4,28,29^

### Treatment durations

Our treatment durations should be viewed with caution because these were usually determined by the standard of care. Nonetheless, the shortest median durations were for injection-free regimens, especially ≥6REZLfx (6.6 months).

### Culture conversion rates

Our overall culture conversion rate and MTCC were comparable to those reported by Salindri et al. and Schechter et al. (overall 75.3% vs. 85.7%, 81.0% and MTCC 33 days vs. 27, 29 days).^10,30^

### Study limitations

Our study has some limitations. A complete record is missing for some patients. The study team collected variables such as sputum results and treatment outcomes according to existing documentation without independent review. We did not routinely document second-line anti-TB drug susceptibility before treatment. However, amplified resistance was uncommon. Not all laboratory-diagnosed TB isolates were tested for INH resistance for operational reasons, e.g., previous algorithms excluded direct Hain for smear-scanty cases and COVID-related laboratory shortages. Because of MSF support, our results may not be transferrable to cohorts where the support provided is not adequate.

## CONCLUSIONS

H^R^R^S^-TB treatment success was high overall. ≥6REZLfx alone had the highest success over the shortest duration; however, the numbers were too small to draw conclusions. Injection-containing regimens were less successful and more likely to result in loss to follow-up. TB programmes should increase their capacity to detect H^R^R^S^-TB, and universal INH screening should be a global goal.
